# WHIM syndrome in a child without the classic tetrad: a case confirmed by de novo CXCR4 mutation

**DOI:** 10.1186/s13223-026-01016-2

**Published:** 2026-02-02

**Authors:** Rayan Al Lohaibi, Khlood Alotibey, Randa Khafaji, Manar Altalhi, Manar Alqahtani, Aryam Alotaibi, Loie Goronfolah

**Affiliations:** 1 Division of Pediatric Allergy Immunology, Department of Pediatrics, King Abdullah Specialized Children’s Hospital, Jeddah, Saudi Arabia; 2 King Abdullah International Medical Research Center, Jeddah, Saudi Arabia; 3 Department of Pediatrics, King Abdullah Specialized Children’s Hospital (KASCH), National Guard Health Affairs, Jeddah, Saudi Arabia; 4 Division of Dermatology, Department of Medicine, Ministry of the National Guard-Health Affairs, Jeddah, Saudi Arabia; 5 Ibn sina national college, Jeddah, Saudi Arabia; 6 College of Medicine, King Saud bin Abdulaziz University for Health Sciences, Jeddah, Saudi Arabia

**Keywords:** WHIM syndrome, CXCR4 mutation, Primary immunodeficiency, De novo mutation, Neutropenia, Pediatric case, Pediatric immunodeficiency

## Abstract

**Background:**

WHIM syndrome is a rare autosomal dominant primary immunodeficiency characterized by the classical tetrad of warts, hypogammaglobulinemia, infections, and myelokathexis. The majority of cases are associated with gain-of-function mutations in the CXCR4 gene. Recent studies have expanded the clinical spectrum of the disease, revealing that only a subset of patients present with all four hallmark features. This underscores the syndrome’s variable expression and the need for greater clinical awareness of its atypical forms.

**Case presentation:**

We report a case of a 6-year-old Saudi girl who presented with persistent neutropenia, recurrent upper respiratory infections, and an episode of thrombocytopenia following a dental procedure. She did not exhibit warts, hypogammaglobulinemia, or myelokathexis. Immunological workup revealed marked lymphopenia affecting T, B, and NK cells, while immunoglobulin levels remained within normal limits. Bone marrow findings were unremarkable. Whole-exome sequencing identified a heterozygous de novo CXCR4 frameshift mutation (c.1172_1173del), confirming the diagnosis of WHIM syndrome. The patient was clinically stable and managed conservatively with precautions.

**Conclusion:**

This case contributes to the evolving understanding of the clinical variability in WHIM syndrome and highlights the importance of genetic testing in patients with unexplained neutropenia and recurrent infections, even in the absence of the complete clinical tetrad.

## Introduction

WHIM syndrome is a rare form of primary immunodeficiency and is considered an autosomal-dominant genetic entity. The acronym WHIM stands for Warts, Hypogammaglobulinemia, Infections, and Myelokathexis, which are considered the classic features of the syndrome. Although myelokathexis is essentially seen in all cases, phenotypic variation exists and has incomplete penetrance [1, 2].

WHIM Syndrome arises from mutations in the CXCR4 gene on human chromosome 2q22 [1]. CXCR4 is a membrane-bound chemokine receptor expressed on many immune cells and hematopoietic stem cells. It is activated by its ligand, CXCL12, which is secreted primarily by bone marrow stromal cells as well as by cells lining blood vessels, neural tissues, and connective tissue. The activation of CXCR4 results in downstream signaling followed by phosphorylation of its C-terminal tail, leading to receptor internalization and signal termination [3].

Its major role is in the trafficking

g and arrest of leukocytes at different specific anatomical sites. It also has a pivotal role in hematopoietic stem cell survival, migration, and homing. The CXCR4 gene mutations lead to a gain-of-function to the CXCR4 receptor, resulting in truncating C-terminal tail protein and failure of signal termination. The CXCL12-CXCR4 hyperactive signaling causes exaggerated retention of leukocytes in the bone marrow and homing of neutrophils from the bloodstream, resulting in robust neutropenia [4, 5].

The first case of WHIM syndrome was reported in 1964 [6]. Since then, approximately 180 cases have been reported in the literature; most were in the United States and Western Europe [2]. It is estimated that 1 out of 4.3 million live births has WHIM syndrome [1]. In Saudi Arabia, there has been only one case report in the literature [7]. Herein, we report a case of WHIM syndrome in Saudi Arabia with a de novo mutation indicated through whole exome sequencing (WES).

## Case report

A 6-year-old Saudi girl presented to the emergency department at King Abdullah Specialized Children’s Hospital (KASCH), Jeddah, with mild pallor and lethargy. Laboratory results from Taif, where she was previously evaluated, showed persistent neutropenia. She had been stable until one week prior when she developed pallor, mild jaundice, and biochemical evidence of compensated hemolysis (reticulocytosis, elevated LDH, and high total bilirubin). On admission, her WBC count was 1.3 × 10^9^/L (reference: 1.5–4) and absolute neutrophil count 0.31 × 10^9^/L (reference: 2–7.5).

She was born full term via cesarean section but required NICU admission for neonatal neutropenia without a clear diagnosis. The family history was negative for consanguinity; both older siblings were healthy. She had received all childhood vaccinations, including MMR and BCG, without adverse reactions. Over the following years, she experienced 6–8 recurrent infections annually, mainly upper respiratory tract and otitis media episodes. At age 5, after a tooth extraction, she developed mucosal hematoma and petechiae, prompting hospitalization in Taif, where she was found to have leukopenia and severe thrombocytopenia (platelets 0 ×10^9^/L). She received platelet transfusion and recovered fully. Subsequent follow-up revealed persistent neutropenia and thrombocytopenia but normal hemoglobin levels.

At KASCH, she was referred to Pediatric hematology, oncology, and immunology. Her G6PD and sickle screen were negative, and hemoglobin electrophoresis was normal. Bone marrow aspiration showed normal cellularity and active trilineage hematopoiesis, without blasts, dysplasia, or hypersegmented neutrophils—confirming peripheral, not marrow, neutropenia.

**Fig. 1:**
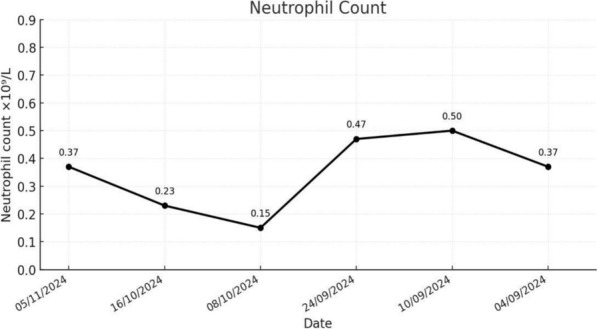
Serial neutrophil count indicating unexplained persistent neutropenia

Immunoglobulin levels were within normal ranges (IgM 1.55 g/L, IgA 1.20 g/L, IgG 8.59 g/L). Nonetheless, the patient also had normal Immunoglobulin G (IgG) subclasses as documented in Table 1**.** Testing confirmed adequate vaccine antibody response. Flow cytometry revealed normal MHC I & II expression but reduced CD3+ T-cells (865 cells/mm^3^), CD8+ T-cells, B-cells, and NK cells demonstrated in Table 2; similar results were noted upon repeat testing**.** Serial blood counts confirmed persistent neutropenia Fig. 1.

**Table 1:** Immunoglobulin G (IgG) subclass levels

ImmUnoglobulin G (IgG) subclasses	Result	Unit	Reference range
IgG 1	7.82	g/l	3.77–11.31
IgG 2	1.96	g/l	0.68–3.88
IgG 3	0.50	g/l	0.16–0.89
IgG 4	0.017	g/l	≤ 1,699

**Table 2:** Flow cytometry analysis of lymphocyte subsets

Subset	% of Lymphocytes	Absolute Count (cells/mm^3^)	Reference range*	Interpretation
CD3 + (Total T Cells)	79%	865	1100–2800	↓ Low
CD3 + CD4 + (T-Helper Cells)	56%	620	500–1800	Normal
CD3 + CD8 + (T-Cytotoxic Cells)	17%	184	400–1200	↓ Low
CD19 + (B Cells)	15%	163	300–700	↓ Low
CD3– CD16 + CD56 + (NK Cells)	6%	64	100–600	↓ Low
CD4/CD8 Ratio	–	3.22	1.0–3.0	↑ Slightly High
Total WBC Count	–	1.6 × 10^9^/L	(4.0–11.0 × 10^9^/L)	↓ Low
Lymphocyte % of WBCs	68.7%	–	–	–

Given these findings, Whole Exome SEQUENCING (WES) was performed for the patient and her parents. WES identified a heterozygous de novo CXCR4 frameshift mutation (NM001348056.2:c.1172_1173del; p.Val320fs)—a pathogenic variant previously linked to WHIM syndrome (ClinVar ID 1067193). Parental WES was negative for this variant, confirming a de novo event. The WES report for the index case as detailed in Table 3 as provided from an external reference laboratory. Following genetic confirmation, the patient was counseled and received the HPV vaccine. She was planned for granulocyte colony-stimulating factor (GCSF) initiation and regular follow-up. As of the last visit, she remains clinically stable with no wart development, although her mother has cutaneous HPV infection.

**Table 3:** Whole Exome Sequencing Report: De Novo CXCR4 Mutation

Gene	Variant coordinates	Amino acid change	SNP Identifier	Zygosity	In silico parameters	Allele frequencies	Type and classification
CXCR4	NM001348056.2:c.1172	p.(Val391Glufs*2	Not	Heterozygous	Polyohen: N/A	gnomAD:-	Frameshift
	_1173del	3)	Applicable		Align-GVDG:	ESP:-	Likely
			(N/A)		N/A	1000 G:-	pathogenic
					SIFT: N/A	CentoMD:-	(Class 2)
					MutationTaster		
					: N/A		
					Conservation_		
					nt:		
					Conservation_		
					aa:		

## Discussion

WHIM syndrome is a rare innate immune disorder with a predisposition to viral infections, especially human papillomavirus (HPV), according to the latest IUIS classification [8].

Mutations in the C-terminal tail Of the CXCR4 Receptor cause retention of myeloid and lymphoid precursors in the bone marrow, leading to peripheral neutropenia seen in nearly all patients and lymphopenia in most [4, 9, 10]. This reflects a high CXCR4 expression on neutrophils whose egress depends on receptor internalization, while lymphocytes rely on other chemokine receptors for egress [11, 12]. CXCR4 also important in lymphocyte maturation, thymic development, and survival [13–15]

The patient's bone marrow biopsy showed normal cellularity without myelokathexis. The degree of marrow retention varies among CXCR4 variants and does not always correlate with leukopenia [16, 17].

Despite the patient's leukopenia, The clinical history was unremarkable for severe infections or recurrent hospitalization. Similar observations suggest that compensatory immune responses and competent leukocytes may reduce infection risk. These mechanisms may explain normal immunoglobulin levels, adequate vaccine response and transient recovery of neutrophil counts during infection [4, 6, 18, 19].

The patient's initial thrombocytopenia resolved after transfusion, suggesting a transient or infectious cause. WHIM patients remain at risk of autoimmune cytopenia, especially in adults. In a cohort study, McDermott et al. reported autoimmune cytopenia in 8 out of 66 patients, while overall autoimmune diseases occurred in 21% [10]. They are also prone to dental complications such as severe periodontitis despite granulocyte colony-stimulating factor therapy [20].

Beyond hematopoiesis, CXCR4 signaling play a role in neuronal and cardiac development [21, 22]. Reported findings include Tetralogy of Fallot, documented in the first reported Saudi case [7], and cerebellar abnormalities [22].

Trio WES revealed a heterozygous de novo CXCR4 mutation in (c.1172_1173del; p.Val391Glufs23), corresponding to (c.959_960delTG; p.Val320fs23) on the MANE transcript, resulting in a frameshift at Val320 and premature stop codon at residue 343 within the C-terminal tail [23].

A similar variant was functionally confirmed by McDermott et al. [10] and included in Zmajkovicova et al. [24] without phenotypic description. García-Carmona et al. describe three related patients with the same variant, all presenting with persistent neutropenia and hypogammaglobulinemia. The two older individuals had recurrent pneumonia and autoimmune cytopenias (ITP, AIHA) treated with steroids and multiple courses of rituximab. One developed inflammatory bowel disease and EBV-related lymphadenopathy, while the other died of sepsis, whereas the youngest presented with lymphopenia and hepatosplenomegaly without autoimmunity and was treated with G-CSF and IVIG [25].

Different receptor internalization defects have been observed among CXCR4 mutations. For example, the nonsense mutation S346 preserves part of the GRK phosphorylation domain, causing mild impairment of receptor internalization, whereas the frameshift mutation S346Pfs*12 produces a truncated and misshaped tail, leading to severe internalization defects [26].

WHIM syndrome follows an autosomal dominant pattern. However, De novo mutations can occur, as seen in our patient, leading to a dominant phenotype even when both parents are neither phenotypically nor genotypically affected. The reported frequencies of de novo, familial, and unknown inheritance across major studies are summarized in Table 4. Germline mosaicism remains possible, as reported by Hernández et al. in two siblings with WHIM syndrome due to a paternal mosaic carrier [1]. The mother however had a normal CBC including neutrophil and lymphocyte counts, with no history of infections or autoimmunity despite her HPV infection, supporting general susceptibility rather than CXCR4 related disorder [10].

**Table 4:** Summary of reported inheritance patterns in WHIM syndrome

Study (year)	Study type	Total number	Denovo n (%)	Familial n (%)	Unknown n (%)
McDermott et al. [10]	International cohort study	66	39 (59%)	20 (30%)	7 (10%)
Méchinaud et al. [30]	Multicenter retrospective cohort	18	12 (67%)	6 (33%)	0
Heusinkveld et al. [4]	Literature review	105	47 (45%)	58 (55%)	0
Hernández et al. [1]	Case series	14	0	12 (86%)	2 (14%)

Targeted therapies such as plerixafor and mavorixafor antagonize CXCR4 signaling in WHIM syndrome. Plerixafor approved for stem cell mobilization and used off-label in WHIM syndrome, appears superior to G-CSF in increasing leukocyte counts and reducing infection risk in clinical trials [27]. Mavorixafor is the first FDA approved treatment for WHIM syndrome with truncating variants [28, 29]. Supportive care includes G-CSF, immunoglobulin replacement, and regular hematologic and immunologic follow-up, with surveillance for HPV and EBV related diseases and malignancies. [4, 30].

## Conclusion

This case expands the known clinical spectrum of WHIM syndrome and the deviation from the classic WHIM tetrad. Further research is needed to identify the systemic effects of CXCR4 mutations and their consequences to support the development of novel therapeutic approaches and management strategies.

## Data Availability

The dataset(s) supporting the conclusions of this article are included within the article. Additional data are not publicly available due to patient privacy and confidentiality considerations.
